# Retraction Statement: Wang, J., Yang, Z., Liu, S., Zhang, L., & Zhao, Y. Silencing of Exosomal miR‐296‐5p Derived from Endothelial Progenitor Cells Enhances Thrombolysis and Recanalization in Deep Vein Thrombosis, *Clin*. *Trans. Sci*. https://ascpt.onlinelibrary.wiley.com/doi/full/10.1111/cts.12799.

**DOI:** 10.1111/cts.13054

**Published:** 2022-02-13

The above article, published online on May 14, 2020 in Wiley Online Library has been retracted by mutual agreement among the authors, the journal Editor‐in‐Chief, Dr. John Wagner, the American Society for Clinical Pharmacology and Therapeutics, and John Wiley & Sons, Inc. The editors had concerns about the validity of the Western blot and qPCR data presented in the article. The authors provided raw data, and software image integrity analysis has confirmed the journal’s suspicions that the Western blots have been inappropriately manipulated.

